# Spectrum of mycobacterial pathogens responsible for head and neck tuberculosis-like presentation

**DOI:** 10.1099/acmi.0.000304

**Published:** 2021-12-17

**Authors:** Kiran Bala, Sanjana Kumari, Rabia Monga, Prem Sagar, Alok Thakar, S. C. Sharma, Urvashi B. Singh

**Affiliations:** ^1^​ Department of Microbiology, All India Institute of Medical Sciences, New Delhi, India; ^2^​ Department of Otolaryngology, All India Institute of Medical Sciences, New Delhi, India

**Keywords:** non-tuberculous mycobacteria, laryngeal tuberculosis, skull base osteomyelitis, maxillary tuberculosis, *Mycobacterium tuberculosis*

## Abstract

Tuberculosis (TB) of the head and neck can be contained in the lymph nodes, larynx, oropharynx, salivary glands, nose and paranasal sinuses, ear, skin and skull. Head and neck TB presentations are varied in nature and thus difficult to diagnose. The clinical features, radiological findings, microbiological diagnostic modalities, surgical and medical management and outcomes of nine cases of head and neck TB are discussed in detail here, together with a thorough review of the literature. Patients presented with atypical symptoms such as discharging sinus, ear lobule swelling, otitis media, vision loss and facial weakness, long refractory otorrhoea and granulation tissue in the ear canal. We diagnosed tubercular skull base osteomyelitis (one case) and laryngeal tuberculosis (two cases), mastoid tuberculosis (one case) and non-tubercular mycobacterial infection involving the temporal bone (two cases), sino-nasal region (one case), maxilla (one cases) and ear lobule (one case) over a period of 8 months. All patients were managed successfully with a combination of surgery and a well-planned treatment regimen for non-tuberculous mycobacteria (NTM) or anti-tubercular drugs for TB. All had successful outcomes except one patient with tubercular skull base osteomyelitis who expired before the initiation of anti-tubercular therapy (ATT). High clinical suspicion followed by thorough diagnostic work-up for both TB and NTM would enable early diagnosis and complete treatment.

## Introduction

According to the World Health Organization (WHO) Global TB report, approximately 10 million people developed tuberculosis (TB) in 2020 and 1.4 million died in 2019 [1]. There were an estimated 1.2 million TB deaths among HIV-negative people in 2019 and an additional 208 000 deaths among HIV-positive people. South-East Asia accounts for 44 % of cases globally and India accounts for 26 % of the burden of TB cases. Two million four hundred thousand TB cases were incident TB cases (new and relapse/recurrent) from India. Tuberculosis affects the pulmonary system (80 % of cases) and extrapulmonary sites in 20 % cases. Head and neck TB constitutes 10–15 % of all EPTB cases, with the majority of these cases being cervical lymph node TB, and <1 % extra nodal head and neck TB cases. Head and neck TB is caused by *Mycobacterium tuberculosis* (MTB; most common) and non-tubercular mycobacteria (NTM; rare). Head and neck TB cases have been reported to involve the cervical lymph nodes, middle ear, larynx, pharynx, paranasal sinus, tonsils and salivary glands [2–4]. The commonest presentation of head and neck TB is tubercular cervical lymphadenopathy. With the lungs being the primary focus, the lymph nodes are involved and a subsequent cough results in the secondary involvement of the larynx, oropharynx and para nasal sinuses [2, 3]. Otomastoiditis due to NTM is extremely rare and has only been described in 56 well-documented cases in the literature in English [2, 5]. Predisposing factors for head and neck TB have been described as poor oral hygiene, local trauma, pre-existing oral leukoplakia, periapical granuloma, periodontitis and nutritional deficiency due to inadequate intake [3]. Clinical presentations of head and neck TB strongly mimic common ENT diseases, with occasional subtle atypical symptoms and signs, which, if missed, lead to misdiagnosis and treatment failure apart from disease progression. Unless strongly suspected, diagnosis of these cases is challenging. We intend to present nine cases of head and neck involvement, including only one case secondary to primary pulmonary TB. Clinical presentation, microbiological diagnostic modalities and treatment outcomes are discussed in detail, together with a thorough review of the literature.

## Subjects and methods

This case series includes patients presenting to the Department of Otorhinolaryngology who were suspected to have tubercular involvement of the head and neck, which was confirmed by the mycobacteriology laboratory in the Department of Microbiology at the All India Institute of Medical Sciences. Detailed demographic findings, unusual symptoms/signs, radiological and histopathological investigations and microbiological tests used for confirmation of diagnosis, presence of primary pulmonary focus, treatment received and treatment response were noted.

### Radiological investigations

All patients underwent thin-section computed tomography (CT) or magnetic resonance imaging (MRI) to evaluate the middle ear and mastoid and temporal bones. Imaging features, including the presence of soft tissue in the middle ear, the integrity of the ossicles and mastoid bony destruction, were analysed and compared with the surgical findings.

### Microbiological investigations

Various samples, such as sputum, pus aspirates and tissue biopsy were collected, depending on the site and the lesion affected in suspected head and neck TB cases. Samples for microscopy and culture were processed using the N-acetyl-l-cysteine-sodium hydroxide method. Decontaminated samples were stained using the Ziehl–Neelsen (ZN) technique and microscopy was performed and graded according to RNTCP guidelines, i.e. scanty (1–9/100 fields), 1+ (10–99/100 fields), 2+ (1–10/field) and 3+ (10/field). Culture was performed using the automated liquid MGIT 960 culture system (BD). Positive cultures were subjected to differentiation into MTB complex and NTM (MGIT TBc Identification test; BD). Positive liquid culture was subjected to MGIT 960 liquid culture drug susceptibility testing. Samples were subjected to molecular detection of TB and rifampicin resistance using the GeneXpert MTB/RIF (Cepheid, Inc.) system and TrueNat MTB-RIF (MolBio, India) in accordance with the manufacturers’ instructions.

The Truenat MTB/NTM assay is a chip-based real-time polymerase chain reaction test for the semiquantitative detection and differentiation of MTB from NTM in human pulmonary and EPTB specimens. Truenat MTB/NTM runs on Truelab Real Time micro-PCR Analyzers. The target genes selected in this assay are the *rpoB* and *nrdz* genes. The *rpoB* gene is a common gene that is present in both MTB and NTM, and its will indicate the presence of total mycobacteria in a given sample. The *nrdz* only specific for MTB and is not present in NTM. The Truenat MTB/NTM test can detect a diverse range of NTM strains. The following NTM strains can be differentiated by the Truenat MTB/NTM assay: *M. avium *, *M. malmoense *, *M. scrofulaceum *, *M. ulcerans *, *M. abscessus *, *M. fortuitum *, *M. gordanae*, *M. szulgai *, *M. kansasii *, *M. asiaticum *, *M. celatum *, *M. simiae *, *M. triviale *, *M. terrae *, *M. flavescens *, *M. haemophilum *, *M. thermoresistibile *, *M. marinum *, *M. xenopi *, *M. vaccae *, *M. chelonae *, *M. smegmatis* and *M. intracellulare *.

## Results

**Fig. 1.**
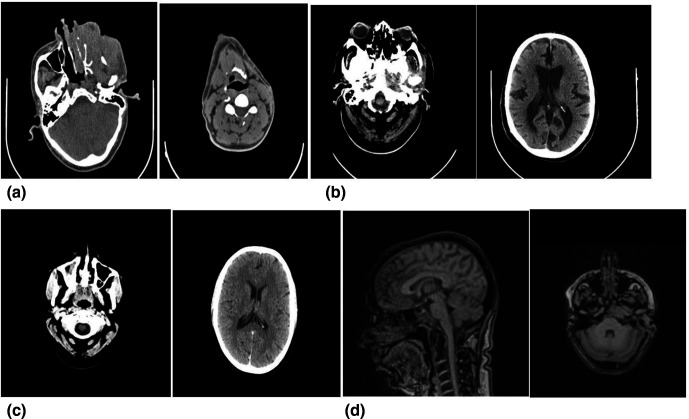
(a) A CT scan of case 1 shows infiltrative lesion of the left maxillary bone. (b) A CT scan of case 3 shows temporal bone soft tissue density – middle ear, mastoid with area of lysis and sclerosis on mandibular condyle. (c) A CT scan of case 5 shows temporal bone soft tissue density filling middle ear and mastoid. (d) A CT scan of case 7 shows post-operative cavity with destruction reaching petrous apex with enhancement along Meckel’s cave and cival region.

A total of nine cases were included over a period of 8 months. Their demographic and clinical details are described in Table 1. Eight patients were adults with an age range from 25 to 85 years and one patient was a 6-year-old child. Seven patients were male and two were female. Two patients had co-morbid conditions; one was on anti-retroviral treatment and the other had diabetes mellitus with hypertension. Patients presented with atypical symptoms such as discharging sinus, ear lobule swelling, otitis media, vision loss and facial weakness, long refractory otorrhoea and granulation tissue in the ear canal. One patient had complained of right vision loss, proptosis and headache for 6 months. One of these nine patients had coexistent pulmonary tuberculosis. None of the patients had a significant past or family history. All patients were suffering for a long span of time and were non-responsive to broad-spectrum antibiotics. External auditory canal, laryngoscopy and audiometry examination showed atypical features that were consistent with tuberculosis or neoplasms. Radiological investigations showed typical features of osteomyelitis of the temporal bone and skull base, and others, such as destruction of bone, increased bone uptake and densities, which needed further evaluation (Table 2, Fig. 1). Histopathological evaluation revealed granuloma in few cases and atypical features in some, suggesting neoplasm. We were able to diagnose involvement of the temporal bone (two cases), tubercular skull base osteomyelitis (one case), mastoid bone (one cases), larynx (two case), sino-nasal (one case), maxillary sinus (1one cases) and ear lobule (one case). Microbiological examination of samples confirmed the cases (Table 2). All samples were acid-fast bacilli-positive (ZN staining), while GeneXpert was positive in four cases for MTB and TrueNat was positive in all nine cases and detected MTB (four cases) and NTM) (five cases (Table 2). All patients were managed successfully with a combination of surgical and combination regimens including antibiotics (NTM cases) and anti-tubercular drugs, except one tubercular skull base osteomyelitis patient, who expired before the initiation of anti-tubercular therapy (ATT) (Table 3).

**Table 1.** Demographic and clinical details of all the patients

Case	Age/ sex	Como rbidi ty	Past H/O of PTB	Complaints	Clinical diagnosis	Clinical feaures/ Atypical if any	Final diagnosis
1	46/M	AIDS (on ART)	No	Left cheek discharging sinus since 3 years	Left maxillary sinus osteomyelitis	Absence of history of trauma/dental extraction, no H/O fever	Maxillary tuberculosis
2	42/M	No	No	Left ear lobule swelling for 6 months	Benign cyst	Cystic swelling with no discharge	Ear lobule tuberculosis
3	82/M	DM and HTN	No	Left ear ache, ipsilateral headache for 3 months, left facial weakness	Skull base osteomyelitis	Congested but intact tympanic membrane, grade 5 left facial weakness	Tubercular skull base osteomyelitis
4	63/M	No	No	Right vision loss and proptosis×6 months; headache ×8 months	Right sphenoethmoid mucocoele	Right visual acquity 6/60 with proptosis	Sinonasal tuberculosis
5	29/F	No	No	Right ear discharge ×3 months, conductive hearing loss (50/20), facial weakness ×2 months, H/O on–off tinnitus and vertigo	Complicated suppurative otitis media	Right tympanic membrane reveals oedematous external auditory canal and mucopurulent discharge with bulge in posterosuperior quadrant; pure tone audiometry revealed right side severe conducting hearing loss with normal left ear; facial weakness grade 5 laryngoscopy WNL	Temporal bone tuberculosis
6	40/M	No	No	Hoarseness ×3 months associated with H/O productive cough	Laryngeal growth with pulmonary tuberculosis	Bilateral true vocal cord and arytenoid irregularity, no H/O smoking /fever/weight loss/loss of appetite	Laryngeal and pulmonary tuberculosis
7	48/F	No	No	Left ear discharge for 3 months (H/O MRM done 1 month back), diplopia for 20 days	Left complicated chronic otitis media with Gradenigo’s syndrome	Florid pale granulations in post-operative mastoid cavity, left mixed hearing loss (55/25), left sixth nerve paresis	Temporal bone tuberculosis
8	42/M	DM	No	Hoarness of voice ×1 year associated with dry cough	Laryngeal TB	Mouse nibbled appearance and ulceration in post-glottis	Laryngeal tuberculosis
9	6/M	No	No	Left ear discharge for 3 months	B/l mastoditis	Granulation tissue on post-EAC wall	Mastoid tuberculosis

AIDS, acquired immunodeficiency syndrome; ART, anti retroviral therapy; DM, diabetes mellitus; EAC, external auditory canal; H/O, history of; HTN, hypertension; MRM, modified radical mastoidectomy; WNL, within normal limits.

**Table 2.** Diagnosis; clinical, radiological, histopathological and microbiological diagnosis

Case	Clinical diagnosis	Radiological investigations	Sample	Histopathological investigations	Microbiological investigations	Organism isolated
Case 1	Left maxillary sinus tubercular osteomyelitis	NCCT PNS – destruction of left maxilla; PETCT – infilterative lesion of left maxillary bone (Fig. 1 a)	Biopsy from maxillary sinus	Markedly hyperplastic stratified squamous epithelium with marked papillomatous and superficial ulcerations; epithelium shows focal mild nuclear atypia	ZN smear – scanty 5–6 bacilli/100 oif; Gene Xpert –negative; TRUNAT- NTM MGIT culture –negative	NTM
Case 2	Benign cyst of ear lobule	None; chest X-ray – WNL	Excision biopsy	Nodular lesion shows acanthotic epidermis; mild chronic inflammatory infiltrate is noted	ZN smear – scanty 4 bacilli/100 oif; Gene Xpert- – negative; TRUNAT-NTM MGIT culture –negative	NTM
Case 3	Skull base osteomylitis	HRCT temporal bone – soft tissue density – middle ear, mastoid with areas of lysis and scleroosis on mandibular condyle. (Fig. 1 b)	Myringotomy done – biopsy from hypertrophic middle ear mucosa	None	ZN smear – 1+; Gene Xpert –not done; TRUNAT-MTB MGIT culture –positive after 3 weeks	MTB
Case 4	Right sphenoethmoid mucocoele	CECT PNS – expansile capsulated lesion involving right posterior ethmoid and sphenoid causing displacement of globe	Endoscopic drainage and tissue biopsy	Inflammatory aetiology	ZN smear – scanty 4 bacilli/100 oif; Gene Xpert – negative; TRUNAT-NTM MGIT culture –negative	NTM
Case 5	Complicated suppurative otitis media	HRCT temporal bone – STD filling middle ear/mastoid; ((Fig. 1 c) bone scan – uptake in right temporal bone; laryngoscopy – WNL	Biopsy sample	Inflammatory aetiology	ZN smear – scanty 3 bacilli/100 oif Gene Xpert –negative; TRUNAT-NTM MGIT culture –negative	NTM
Case 6	Laryngeal and pulmonary tuberculosis	X-ray chest – left upper lobe consolidation; laryngoscopic examination reveals bilateral irregular cords with granular lesion	Laryngeal biopsy and sputum sample	Direct laryngoscopic biopsy showed epitheloid cell granuloma with langhan type giant cell consistent with tuberculosis	ZN smear – scanty 2–3 bacilli/100 oif; Gene Xpert – positive; TRUNAT- MTB MGIT culture –negative	MTB
Case 7	Left gradenigo syndrome	HRCT temporal bone and CEMRI shows post-op cavity with destruction reaching petrous apex with enhancement along Meckel’s cave and clival region; (Fig. 1 d) X-ray chest – WNL	Biopsy from cavity granulation	Inflammatory granulation tissue and occasional bone fragments, no necrosis or dysplasia seen	ZN smear – scanty 3 bacilli/100 oif; Gene Xpert –negative; TRUNAT- NTM MGIT culture –negative	NTM
Case 8	Laryngeal TB	CECT neck shows diifuse mucosal thickening in B/L maxillary sinus and ethmoid sinus	Biopsy from larynx	Poorly differentiated malignant tumour S/O non-Hodgkin lymphoma	ZN smear – scanty; Gene Xpert – positive; TRUNAT – MTB; MGIT culture –negative	MTB
Case 9	Mastoid tuberculosis	No	Pus from ear	No	ZN smear – scanty 2–3 bacilli/100 oif; Gene Xpert –positive; TRUNAT – MTB; MGIT culture –negative	MTB

B/L, bilateral; CEMRI, contrast enhanced magnetic resonance imaging; HRCT, high resonance computed tomography; MGIT, mycobacterium growth indicator tube; MTB, *Mycobacterium tuberculosis *; NTM, non-tubercular mycobacteria; S/O, suggestive of; STD, soft tissue densities; WNL, within limits; ZN, Ziehl–Neelsen.

**Table 3.** Medical and surgical management and outcome for all patients

Serial no.	Age/sex	Final diagnosis	Medical treatment	Surgical treatment	Response to treatment
1	46/M	Maxillary tuberculosis	ATT CATEGORY 1 9 months	Surgical debridement from maxillary sinus	Cured Healed scar present over left cheek Follow-up done for 18 months; no discharge
2	42/M	Ear lobule tuberculosis	ATT CATEGORY 1 6 months	Excision biopsy with primary suturing	Cured Follow-up done for 7 months Well-healed local site
3	82/M	Tubercular skull base osteomyelitis	Patient expired before start of ATT due to diabetic nephropathy	Myringotomy done under LA	Expired
4	63/M	Sinonasal tuberculosis	ATT CATEGORY 1 6 months	Endoscopic drainage and tissue debridement	Cured Right eye vision improved (6/18) No proptosis Well-healed sinonasal cavity
5	29/F	Temporal bone tuberculosis	IV ceftazidime and amikacin ×4 days (amikacin discontinued due to ototoxicity) I/V imipenem and oral clarithromycin, levofloxacin ×7 days (oral clarithromycin, levofloxacin, linezolid ×6 months)	Cortical mastoidectomy with tympanoplasty placement	Cured No ear discharge for 9 months Facial nerve function improved – grade 3, intact Tympanic membrane
6	40/M	Laryngeal and pulmonary tuberculosis	ATT CATEGORY 1 6 months	Direct laryngoscopic biopsy.	Cured Follow-up for 1 year Voice quality improved markedly Laryngeal evaluation reveals no lesions over cords; only mild congestion seen
7	48/F	Temporal bone tuberculosis	ATT CATEGORY 1 with tab clarithromycin 500 mg BD 12 months	Cortical mastoidectomy with tympanoplasty placement	Cured Lateral rectus function improvement after 2 weeks of ATT Well-healed mastoid cavity with no granulations at present
8	42/M	Laryngeal TB	ATT CATEGORY 1 6 months	Direct laryngoscopic biopsy	Cured Follow-up for 1 year Voice quality improved markedly
9	6/M	Mastoid tuberculosis	Tab rifacept – kid 3 od Tab ethambutol 400 mg od	No	Cured; follow-up is ongoing

ATT, anti tubercular treatment; BD, twice a day; I/V, intravenous; OD, once a day.

## Discussion

Ample published literature has discussed and described head and neck TB [2]. Non-tuberculous mycobacteria (NTM) have also been reported to cause head and neck involvement. NTM are ubiquitous micro-organisms in natural and constructed environments. NTM are known to cause a broad spectrum of diseases, including lymphadenitis and skin, soft tissue, ocular, abdominal, pulmonary, wound and disseminated infections. Tubercular cervical lymphadenopathy is the commonest presentation and is characterized by sub-acute to chronic or recurrent lymphadenopathy, usually bilateral, painless, with or without intermittently discharging sinuses leaving scars [2]. Common presented symptoms incude low-grade fever episodes, loss of appetite, weight loss and occasionally history of close contact with patients of TB. Most of the head and neck TB cases have evidence of pulmonary disease and secondary infection in the larynx and nose as well as paranasal sinuses through coughing and sneezing infected material through [2–4]. Secondary aural infection occurs through seeding of the middle ear by nasopharyngeal spread or by the passage of infected material up the auditory tube through coughing. Haematogenous implantation can lead to the spread of mycobacterial infection, potentially to any site of the body, including the head and neck. Pulmonary or systemic TB must be investigated to exclude it from head and neck TB patients. In our case series, only one laryngeal tuberculosis patient had co-existing active pulmonary tuberculosis, a direct inoculation of mycobacterium from the air inhaled through the eustachian tube or haematogenous inoculation could be some of the possible routes of infection. A healed primary pulmonary focus (with so many commonly prescribed antibiotics being effective against TB) but persistent bony involvement in temporal bone or a reactivation of an old healed focus in the airways and localizing in the head and neck region are a few other possibilities. The continuum of airways from alveoli to the inner ear would play host to all airborne pathogens. The site of localization of the disease and hence the disease presentation could vary. Although there is more awareness of pulmonary TB, the importance of suspecting the disease in the head and neck airways needs to be understood.

In a retrospective analysis of 117 patients with primary TB of head and neck, Bhat *et al*., reported 95 % patients with cervical lymphadenopathy, two cases with laryngeal TB, and one each with TB of cervical spine, oropharynx, ear and retropharyngeal abscess [2]. In our study, we diagnosed seven cases of head and neck TB over a period of 8 months; most of them did not present with the most commonly reported tubercular cervical lymphadenopathy. Instead, we found temporal bone TB (two cases), tubercular skull base osteomyelitis (one case), laryngeal TB (one case), sino-nasal TB (two cases) and ear lobule TB (one case).

Tuberculosis of the middle ear and mastoid presents with persistent painless otorrhoea, otalgia, hearing loss and, in extreme cases, facial palsy. TOM (tuberculous otitis media) has a typical presentation of multiple tympanic membrane perforations [4, 6]. Thin mucoid or sanguineous otorrhoea and abundant pale polypoid tissue have been seen during otological examination [4, 6]. Radiology shows a background of pneumatized mastoid and features of bony involvement and sequestrum formation. Tubercular otitis media/mastoiditis should be suspected when signs suggestive of extensive disease as compared to minimal symptoms. Biopsies from the middle ear or mastoid granulations are supportive for confirming the diagnosis.

Tuberculosis of the nasal and the paranasal sinuses presents with non-specific symptoms such as nasal obstruction and blood-stained rhinorrhoea and headache, with mimicking as sinusitis and neoplastic lesions. Tubercular suspicion becomes stronger when signs of recurrent discharging sinuses over the cheek or zygomatic area are evident [5, 7]. Detailed clinical endoscopic examination and biopsies from the pale granular tissue are the steps towards making a diagnosis. One case of maxillary tuberculosis presented with persistent discharging sinus for 3 years. The patient was known to have AIDS and was on antiretroviral therapy. One case of sino-nasal tuberculosis presented with atypical features, such as right-sided vision loss (visual acquity, 6/60) with proptosis for 6 months and headache for 8 months.

The case of ear lobule tuberculosis had very unusual features, and presented with a benign cyst of the ear lobule without discharge for 6 months or any history of trauma or ear piercing. A case report involving a 62-year-old woman with right pinna tuberculosis, diagnosed using histopathology and confirmed using microbiological methods, was treated successfully with ATT [7]. Another case report of Tuberculosis verrucosa cutis is recorded for a young woman with keloid over ear lobule (the keloid increased in size over 4 months) [8]. We diagnosed an unusual causative organism in our patient, an NTM identified as acid-fast bacilli microscopically and confirmed by Truenat. This patient was treated with a combination regimen including several antibiotics.

Laryngeal tuberculosis has been reported to be the second most commonly reported TB in the head and neck region followed by cervical lymphadenitis. Co-infection of pulmonary tuberculosis is found in ~1 % of laryngeal tuberculosis cases. Laryngeal TB is commonly associated with an undiagnosed, inadequately treated or recurrent pulmonary tuberculosis. Our laryngeal TB case also had a co-existing active primary pulmonary tuberculosis. Presenting symptoms include dysphonia, cough, odynophagia, dyspnoea, dysphagia, hoarseness of voice and weight loss [9]. Patients undergoing direct laryngoscopy and biopsy have laryngeal TB detected in 6.25 % of cases. Indirect laryngoscopy/fibroptic laryngoscopic examination commonly reveals diffuse pale and polypoidal thickening of the epiglottis, vocal cords and arytenoids, closely mimicking neoplastic lesions and requires pathological as well as microbiological evaluation to confirm the diagnosis [10]. The laryngeal tuberculosis case in our series presented with hoarseness of voice in the last 3 months, associated with a productive cough.

Skull base osteomyelitis (SBO) has been reported as a complication of otitis externa due to *Pseudomonas aeruginosa* in older diabetic patients and most commonly involves the temporal bone. Atypical or central skull base osteomyelitis arises from the sphenoid or occipital bones rather than the temporal bone and is not usually associated with otitis externa [11, 12]. Incidence of atypical SBO has been reported much less frequently and initially presents with headache as the only symptom, with cranial neuropathies occurring in the later course of disease. The published literature reveals that small-vessel disease of the external auditory canal (EAC) and an increase in the cerumen pH in diabetic persons can predispose patients to a rapid and at times devastating disease course [13]. When infection traverses the fissures of Santorini and the tympanmastoid suture to the Haversian system of the compact bone, and finally to the skull base, then SBO can lead to cranial nerve (CN) palsies. CN VII is most commonly affected, due to the proximity to the EAC on exiting the skull base, followed by CNs IX, X, XI and XII [14, 15]. Our case was a classic case of tubercular skull base osteomyelitis in a 82-year-old diabetic and hypertensive who presented with otorrhoea, ear ache and headache with grade 5 left facial weakness and succumbed to the disease before the initiation of antitubercular therapy.

Computed tomography is helpful in making a diagnosis and to evaluate the extent and severity of extratemporal soft tissue involvement and skull base osteomyelitis. Soft tissue inflammation, usually seen/limited in the early stages of disease and advanced disease, involves skull base bone destruction and abscess formation. The HRCT findings in our case were consistent with those reported in advanced disease [14].

Temporal bone osteomyelitis (TBO) occurs as a complication from necrotizing external otitis media (NEO) and middle ear infection. TBO is a rare but very aggressive disease with different aetiologies [16]. TBO predisposes patients to clinical conditions such as trauma, bone surgery, or other diseases that can affect the vascularity of bone (osteoporosis, osteopetrosis, Paget’s disease, radiation and malignancy) [17]. Facial neuropathy is most commonly reported and facial paralysis occurs in 25 % of patients due to the involvement of the stylomastoid foramen [18, 19]. A case of local temporal bone osteomyelitis caused by granulomatous tuberculous infection has been reported [20]. Two of our patients were suffering from complicated suppurative chronic otitis media and presented with otorrhoea, conductive hearing loss, facial weakness and diplopia (in one case). The radiological findings in both the cases confirmed them as temporal bone osteomyelitis and microbiological examination detected NTM in both cases, guiding treatment decisions (as per the ATS guidelines, 2007) [23] and securing a favourable outcome. Yeh *et al*. discussed the fact that NTM otomastoiditis with TBO should be suspected in cases of chronic otorrhoea, otalgia and granulation tissue in the ear [21].

Few cases of NTM otomastoiditis have been reported in the published literature. A retrospective case series has reported the rates of granulation tissue (90.9 %), otalgia (31.8 %), facial palsy (9.1 %) and lung involvement (5.4 %). Cases of temporal bone osteomyelitis have been reported, most commonly with the NTM *M. abscessus* (52.7 % of cases) and *M. avium-intracellulare*, followed by *M. fortuitum* [21–23]. Three distinct routes have been suggested for the entry of NTM into the middle ear: direct inoculation through perforation of the tympanic membrane or a tympanostomy tube (most common), through the eustachian tube, and direct spread via improperly sterilized surgical instruments [22, 23]. We detected five patients with NTM. Two patients had NTM temporal bone osteomyelitis, and had presented with otalgia, otorrhoea, facial palsy and granulation tissue and previous history of tympanic membrane perforation. Gardenigo syndrome caused by NTM has also been reported [24]. Our case fitted the definition of Gardenigo syndrome, diagnosed as temporal bone osteomyelitis caused by NTM.

Head and neck TB (MTB and NTM) remains a diagnostic and therapeutic challenge for clinicians due to varied presentation. Multiple diagnostic modalities aided to the accurate diagnosis and treatment. The published literature and ATS guidelines suggested a macrolide drug (clarithromycin) to be the most appropriate initial regimen with a combination of parenteral amikacin and/or imipenem for treatment of NTM [23]. The duration of antibiotic use depends on response to treatment for each case, but is at least 6 months with careful monitoring, and longer if required. Long-term multiple antibiotic therapy is advised for at least 1 to 3 months after the symptoms have resolved. NTM have a tendency to reproduce and exist in biofilms, which makes them difficult to destroy [21, 23]. Surgical debridement or even repeated surgery with antibiotic therapy to remove the NTM and the surrounding biofilms is required to accomplish infection control and efficiently manage such infections.

## Conclusion

Clinicians should maintain a high suspicion for head and neck TB. Unusual presentations such as refractory otorrhoea, granulation tissue in the middle ear cavity and mastoid air cells, or cortical bone or ossicular chain destruction on imaging without evidence of a cholesteatoma suggests a need for head and neck TB work-up. Whenever accurate diagnosis has been confirmed, surgical and medical intervention (well-designed regimen) should be implemented and a symptom-free course should be ensured before antibiotics are stopped.
